# Can hypnotic suggestibility be measured online?

**DOI:** 10.1007/s00426-019-01162-w

**Published:** 2019-03-05

**Authors:** Bence Palfi, G. Moga, P. Lush, R. B. Scott, Z. Dienes

**Affiliations:** 1 School of Psychology, University of Sussex, Brighton, UK; 2 Sackler Centre for Consciousness Science, University of Sussex, Brighton, UK; 3 School of Informatics, University of Sussex, Brighton, UK

## Abstract

Hypnosis and hypnotic suggestions are gradually gaining popularity within the consciousness community as established tools for the experimental manipulation of illusions of involuntariness, hallucinations and delusions. However, hypnosis is still far from being a widespread instrument; a crucial hindrance to taking it up is the amount of time needed to invest in identifying people high and low in responsiveness to suggestion. In this study, we introduced an online assessment of hypnotic response and estimated the extent to which the scores and psychometric properties of an online screening differ from an offline one. We propose that the online screening of hypnotic response is viable as it reduces the level of responsiveness only by a slight extent. The application of online screening may prompt researchers to run large-scale studies with more heterogeneous samples, which would help researchers to overcome some of the issues underlying the current replication crisis in psychology.

## Introduction

Hypnosis and hypnotic suggestions have been shown to be useful experimental tools to test theories of cognitive neuroscience (Oakley & Halligan, 2013; Raz, 2011), especially theories related to consciousness (Cardeña, 2014; Terhune, Cleeremans, Raz, & Lynn, 2017). For instance, hypnotic suggestions can evoke changes in the feeling of voluntariness (Weitzenhoffer, 1974, 1980) or modify one`s sense of agency (Haggard, Cartledge, Dafydd, & Oakley, 2004; Lush et al., 2017; Polito, Barnier, & Woody, 2013). Responses to suggestions frequently involve alterations in perception, such as the experience of positive and negative hallucinations or delusions (Kihlstrom, 1985; Oakley & Halligan, 2009). Moreover, hypnotic suggestions can be employed to simulate some properties of neurological and psychiatric conditions in healthy subjects (Barnier & McConkey, 2003; Oakley, 2006). Finally, correlations between hypnotisability and measures employed by consciousness researchers (e.g. the rubber hand illusion; the vicarious pain questionnaire; mirror touch synaesthesia) have recently been found (Lush et al., 2019). These correlations suggest that measures common in the consciousness literature are driven by hypnotic suggestibility. There is therefore an increasing need for an expansion of hypnosis research. Unfortunately, the successful application of hypnotic suggestions demands plenty of resources, making it impractical for researchers to run large-scale hypnosis related studies. In order to conduct experiments involving hypnosis, researchers generally need to recruit from a specific subsample of people based on their tendency to respond to hypnotic suggestions. To achieve this, researchers run hypnosis screening sessions before recruitment, so that, for example, they can identify the participants at the lowest and highest end of the scale (low and highly hypnotisable people, respectively). High and low hypnotisability are usually defined as the top and bottom 10–15% of screening scores (Barnier & McConkey, 2004; Anlló, Becchio & Sackur, 2017). Therefore, screening procedures are time-consuming; to identify a single highly suggestible participant for an experiment, one has to find, on average, ten people who are willing to undertake a screening that can last from 40 up to 90 min depending on the applied method.

The hypnosis screening procedure has moved through a long developmental process in which it has become more and more user friendly. Initially, the screening consisted of two steps, a preliminary group session applying the Harvard Group Scale of Hypnotic Susceptibility Form A (HGSHS:A; Shor & Orne, 1963) and an individual session using the Stanford Hypnotic Susceptibility Scale Form C (SHSS:C; Weitzenhoffer & Hilgard, 1962) conducted with only those scoring very high or low in the first session. The later development of a reliable group screening method, the Waterloo-Stanford Group Scale of Hypnotic Susceptibility (WSGC; Bowers, 1993), has drastically mitigated the time required for screening as it allows researcher to screen up to a dozen people in about 90 min (although it was originally intended to act as a second screen after an HGSH:A, a single screen with the WSGC is quite reliable enough to select subjects capable of later having compelling subjective responses to difficult suggestions, e.g. digit–colour synesthesia, Anderson, Seth, Dienes, & Ward, 2014, or compelling objective reductions in Stroop interference to alexia (word blindness) suggestions, e.g. Parris, Dienes, Bate, & Gothard, 2014). Recently, the Sussex Waterloo Scale of Hypnotizability (SWASH; Lush, Moga, McLatchie & Dienes, 2018) was introduced, which is a modified version of the WSGC. The SWASH includes new items to measure the subjective experiences of the participants (compare also the Carleton University Responsiveness to Suggestion Scale [CURSS, Spanos, Radtke, Hodgins, Stam, & Bertrand, 1983], the Creative Imagination Scale [CIS, Wilson & Barber, 1978], and the Experiential Scale for the WSGC [Kirsch, Milling, & Burgess, 1988]). The length of the procedure was reduced to 40 min and it can be run with larger groups than the WSGC (Lush et al., 2018). Moreover, the dream and age regression suggestions were not included in the SWASH. These highly personalised items of the WSGC can be risky by virtue of possibly triggering unpleasant memories or emotions (Cardeña & Terhune, 2009; Hilgard, 1974).

Nonetheless, the application of the least demanding methods (such as the SWASH, the CURSS or the CIS), still requires potential participants to attend a group session, which makes the screening procedure relatively time-consuming and limits the subject pools to psychology students who are the easiest to incentivise to participate in a group screening on campuses. These two barriers of large-scale hypnosis studies could be overcome by employing fully automatised, online hypnosis screening procedures. In the last two decades, psychological science has witnessed growth in the application of online data collection for experimental purposes, paving the way for researchers to collect large samples in a short period of time (Reips, 2000; though it can come with its own problems, e.g. Dennis, Goodson & Pearson, 2018). In order to adapt the hypnosis screening procedure online, one needs to ensure that the non “live” version can induce similar objective and subjective hypnotic responses as with a “live” hypnotist. Indeed, suggestibility scores of participants are comparable when the hypnotic induction and suggestions are delivered by a pre-recorded audiotape and when they are delivered by an experimenter (Barber & Calverley, 1964; Fassler, Lynn, & Knox, 2008; Lush, Scott, Moga, & Dienes, 2019). These findings underpin the idea that the participants could easily undergo a hypnosis screening procedure in their own rooms by listening to a pre-recorded script and filling out the booklets online. Nevertheless, online data collection has its own perils, namely, the data acquired by online questionnaires might not be as reliable and the results might not be consistent with the ones of the traditional data collection procedures (Krantz & Dalal, 2000). Therefore, the reliability of new online questionnaires, such as the online version of a hypnosis screening procedure, needs to be tested even if there is evidence that the quality of the data and the findings of online-based studies can be similar to those obtained by traditional methods (Gosling, Vazire, Srivastava, & John, 2004; Buhrmester, Kwang, & Gosling, 2011).

In this project, our purpose is to explore the extent to which an online hypnotic screening procedure is reliable and consistent with an offline procedure. To this aim, we measured people`s hypnotic suggestibility with the SWASH on two separate occasions and in two different environments. Henceforth, we call every type of data collection carried out in a controlled environment with the experimenter present an offline screening, whereas undertaking a hypnotic screening alone in one’s own room under one’s own control will be called online screening. In addition, we are interested in the extent to which the length of the delay between first and second screen can influence the reliability and the scores of hypnotic suggestibility. The question about the stability of hypnotic suggestibility over periods of few days or even decades have inspired various research projects (e.g. Fassler, Lynn, & Knox, 2008; Lynn, Weekes, Matyi, & Neufeld, 1988; Piccione, Hilgard, & Zimbardo, 1989). To assess the stability of hypnotic suggestibility, we recruited half of the sample from the subject pool of the year of 2016 and the other half from the year of 2017, both of whom have already received offline screening. Therefore, for some of the participants, the delay between the two screenings is not more than 6 months (short delay group), whereas for the others, it is at least one and a half years (long delay group). For practical reasons, the first screening was organised offline, in groups of 20–40 for all the participants, whereas the second screening was either an online screening or another offline one. By this method, we are able to estimate how strongly the type of the screening and the length of the delay can influence the suggestibility scores of the people; we can also assess their influence on the test–retest reliability and the validity of the screening. Taken together, this project strives to explore whether a well-established offline screening procedure could be replaced for practical purposes by an online version, which could help consciousness researchers run more and larger hypnosis studies by drastically cutting the recruitment-related costs.

While responding to hypnotic suggestions, people tend to experience as of being in some form of trance or altered state (Kihlstrom, 2005; Kirsch, 2011). This experience is usually measured by subjective reports of depth of hypnosis (e.g. Hilgard & Tart, 1966), which is, interestingly, strongly associated with people`s ability to respond to hypnotic suggestions (Wagstaff, Cole, & Brunas-Wagstaff, 2008). We investigate this link by assessing the strength of relationship between hypnotic suggestibility scores and depth of hypnosis reports, and the extent to which the mentioned experimental manipulations can influence this relationship. We also aim to evaluate the extent to which depth of hypnosis is influenced by the type of data collection and the length of the delay between screens to ensure that people experience comparable level of hypnotic depth during online and offline screens.

In our analyses, we solely employed estimation procedures instead of testing the existence of differences with an inferential statistical tool such as the null-hypothesis significance test (Fisher, 1925; Neyman & Pearson, 1933) or the Bayes factor (e.g. Dienes, 2011; Rouder et al., 2009). Estimation is recommended over inferential statistics when the existence of a difference is established or it is not relevant (Jeffreys 1961; Wagenmakers et al., 2018). The second point proves to be decisive for our case, since it is not necessary to test the existence of any investigated effect to answer our research questions. For instance, the core aim of the current project was to conclude regarding the applicability of online hypnosis screening by comparing the SWASH scores, the reliability and the validity of online and offline hypnosis screening. Imagine a scenario in which an inferential statistical tool demonstrates evidence for the difference between the offline and online groups in favour of the offline group in all aspects that assess the quality of the measurement. Importantly, this outcome per se cannot give a definite answer to our central question as the mere fact that offline screening is significantly better than online screening neglects the question of magnitude of the difference. To reject or accept the idea that online screening is viable, we need to know the extent to which the quality of offline and online screening differs so that we can decide whether the benefits of the online screen outbalance its costs. Further, the fact that the two types of screening will correlate cannot be in doubt; the question is simply the strength of the relationship between them.

To explore the range of plausible effect sizes, estimation methods, either from the Bayesian (Kruschke, 2010, 2013; Rouder, Lu, Speckman, Sun, & Jiang, 2005; Wagenmakers, Morey, & Lee, 2016) or from the frequentist school (Cumming, 2014) can be used. Here, we applied a Bayesian tool, estimation by calculating the 95% Bayesian Credibility Intervals, as this is the method that is appropriate to answer our research question; namely, how confident can we be that the true effect size lies within a specific interval (Wagenmakers et al., 2018). Only Credibility Intervals allow us to make claims such as that the true value of the effect size is probably not larger or smaller than a particular value.

## Methods

### Participants

Psychology students at the University of Sussex participated in an offline hypnosis screening as part one of their modules during the first semester of their studies. We recruited psychology students who had started their BSc studies in the year of 2016 or 2017 and who had provided their contact information in an offline hypnosis screening session. Both subject pools consisted of around 300 students and we randomly assigned half of them to each experimental group (experimental groups described below). Thus, we invited around 150 people for each group. We continued data collection until the end of the spring semester of 2018. In the second session, 73 students participated. However, we could not trace back the data of two students to their first session results and so we needed to exclude them from all of the analyses, leaving us with 71 participants in total. Twenty-six students attended the offline session (23 females, *M*_age_ = 19.7, SD_age_ = 1.8) and 45 students completed the screening online (41 females, *M*_age_ = 21.0, SD_age_ = 5.3).

We informed each participant about the nature of the study and only those students were be able to attend who agreed to the terms and conditions of the study. After finishing the experiment, the participants were debriefed and received a payment of £5 or course credit. The study has been approved by the Ethical Committee of the University of Sussex (Sciences & Technology C-REC).

### Materials

One of the authors produced the audio recording of the hypnosis procedure (induction and the suggestions); the length of this recording was 28 min. The questionnaire applied in the first session for data collection was created in MatLab (MathWorks, 2016), whereas the questionnaire that was used in the second session was a PHP-based website. The PHP script, the materials and the documentation on how to instal the software can be accessed at https://osf.io/6twdp/.

### Measures

The measures introduced below were utilised in the first occasion of the data collection. The second occasion only included the assessment of the hypnotic suggestibility measured by the SWASH regardless of the type of the session (offline or online). Note that, although several questionnaires were registered along with the first screening, we only used the suggestibility scores of the participants in this project (see our research questions in the last paragraph of the Introduction).

#### SWASH

The hypnotisability of the students was measured by the SWASH. This scale is a modified version of the WSGC (Bowers, 1993) which contains 10 suggestions and corresponding items measuring objective suggestibility and the subjective experiences of the participants about each suggestion.

#### Data collection in 2016

As part of the first session in 2016 the following four questionnaires were registered: (a) Barratt Impulsiveness Scale (BIS-11), which consists of 30 items and measures people`s tendency to behave impulsively (Patton & Stanford, 1995); (b) Free Will Inventory (FWI), which includes 29 items measuring people`s beliefs about free will and their relationships with these beliefs (Nadelhoffer, Shepard, Nahmias, Sripada, & Ross, 2014); (c) Short Form of the Five Facet Mindfulness Questionnaire (FFMQ-SF), which is a 24-item scale assessing the mindfulness skills of individuals via self-report (Bohlmeijer, ten Klooster, Fledderus, Veehof, & Baer, 2011); (d) Dissociative Experiences Scale-II (DES-II), which is a 28-item self-report questionnaire developed by Bernstein and Putman (1986).

#### Data collection in 2017

In 2017, we administered the following four questionnaires in the first data collection session of: (a) a 15-min-long breath counting exercises based on Study 2 of Levinson, Stoll, Kindy, Merry & Davidson (2014); (b) the Mindful Attention Awareness Scale (MAAS) consisting 15 Likert scale items (Brown & Ryan, 2003); (c) the Schizotypal Personality Questionnaire-Brief (SPQ-B), which consists of dichotomous questions (Raine & Benishay, 1995); (d) the DES-II that was used in 2016.

### Design

We employed a 2 × 2 × 2 mixed design. The within subject variable is the date of the data collection (first session vs. second session). The between subjects independent variables are the form of the second hypnosis screening session (offline vs. online) and the length of the delay between the first and the second sessions (short delay [few months] vs. long delay [more than a year]).

### Procedure

There were three forms of data collection: (1) group sessions at the university with the experimenter present (first, offline screen); (2) individual sessions in a small experimental room at the university with the experimenter present (second, offline screen); (3) individual sessions at home (second, online screen). All of the participants engaged in the first, offline, screen and later they were invited to attend in a second screen that was either offline or online. The procedure of the screening was identical in each case and followed the steps below.

After providing informed consent, the participants had the opportunity to provide contact details for a database in case they were willing to participate in hypnosis related research in the future. Next, they were asked to adjust the volume of their headphones until it was moderately loud by listening to a test tune. Before starting the hypnotic induction procedure, they were notified that the whole procedure would last about 45 min and that they should not take a break. By pressing the start button, participants ran the hypnotic induction and suggestions. After the de-induction, participants were asked to fill out the SWASH response booklet, rating their response to each suggestion. Finally, the participants were thanked for attending and debriefed.

### Data analysis

#### Data transformation

**Fig. 1:**
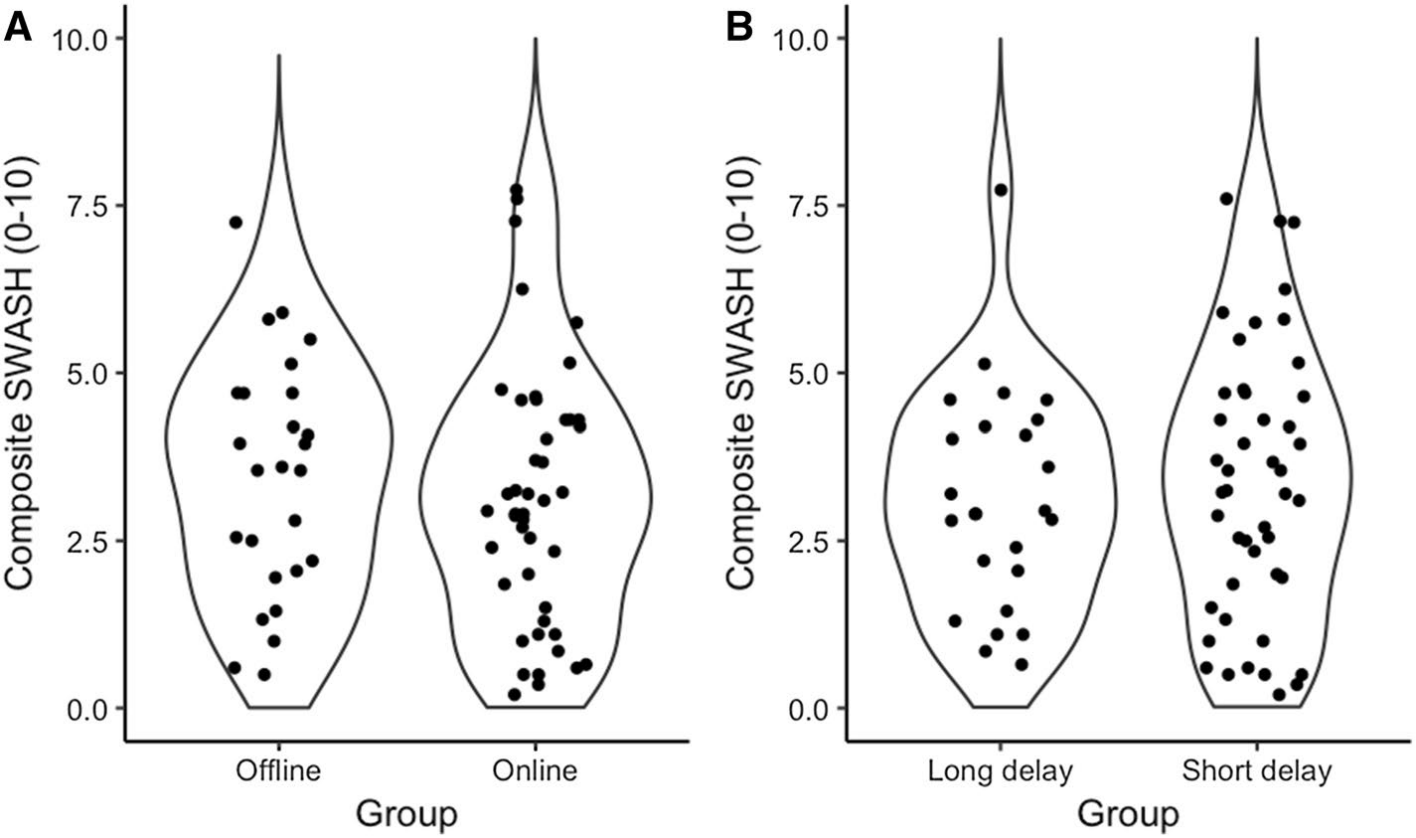
Violin plots depicting the distribution of composite SWASH scores of the second screens broken down either by the type of the screen (offline vs. online, **a**) or by the length of the delay (long vs. short delay, **b**). Each black dot indicates a composite SWASH score of a participant

We computed the Objective and Subjective suggestibility scores of the participants as described in the SWASH manual (Lush et al., 2018) and then we doubled all subjective scores so that both of the objective and subjective scores fell between 0 and 10. By taking the weighted average of these derived scores, we calculated the composite SWASH score of each participant, which was used in the majority of the analyses. For more details on the calculation of the SWASH scores, see Lush et al. (2018; manual available at https://osf.io/wujk8/). Given that the distributions of the objective, subjective and composite SWASH scores of the first screen were all fairly normal (see Fig. 1 in Preregistration), we assumed that the dataset of the second screen was also normally distributed. Therefore, we planned to use parametric methods to estimate the strength of correlation between continuous variables (Pearson`s *r*).

#### Bayesian estimation

In this project, we estimated the population effect sizes and did not test hypotheses. Thus, here, we report the estimates (e.g. mean or correlation) and the 95% Bayesian credibility intervals (CI) applying a uniform prior distribution. Note that, although, the bounds of the CIs are numerically equal to the bounds of the confidence intervals (assuming a uniform prior), their interpretation is different (e.g. Morey, Hoekstra, Rouder, Lee, & Wagenmakers, 2016).

### Implementation of the preregistration

The design and research questions of this study were preregistered at osf.io/3abje. In order to ensure the reproducibility of the analysis and decrease analytic flexibility, we preregistered an analysis script, written in R (R Core Team, 2018), a prior to data collection. The script includes all of the steps defined in the preregistration and an additional data simulation, which helped us test and debug the script. In this paper, we present the results of analyses that were preregistered in the above-mentioned R script and results of two additional, non-preregistered analyses: (1) test–retest reliability of SWASH scores; (2) correlation between SWASH and depth of hypnosis scores. We deviated from the analysis script in one aspect. The calculation of the 95% CIs of the differences between two correlations was incorrect in the original script due to an issue with back-transformation of Fisher`s *z* values of difference scores to Pearson`s *r* (e.g. Meng, Rosenthal & Rubin, 1992; Olkin & Finn, 1995). Therefore, we used the cocor R package (Diedenhofen & Musch, 2015), which is based on the approximation method of Zou (2007), to estimate the 95% CIs of the differences between correlations.

## Results

### SWASH scores

The mean of the composite SWASH scores in the offline group (*M* = 3.44) was only slightly larger than the mean of the online group (*M* = 3.13) rendering their difference negligible (*M*_diff_ = 0.31, 95% CI [-0.59, 1.22]). Crucially, the difference between the groups is unlikely to be larger than 1.22. The difference between the offline and online groups is likely to be negligible or small for both of the objective (*M*_diff_ = 0.39, 95% CI [− 0.58, 1.36]) and subjective subscales (*M*_diff_ = 0.24, 95% CI [− 0.72, 1.19]). Panel A of Fig. 1 demonstrates that the distribution of the composite SWASH scores of the offline group is akin to the online group. The density of the data is similar between the groups even around the right tail (top) of the distribution indicating that similar proportion of the participants scored high on the SWASH in the offline and online groups. The mean of the composite SWASH scores was comparable in the short (*M* = 3.32) and long delay groups (*M* = 3.10), and the plausible values of their differences vary around zero with a maximum difference of 1.10 (*M*_diff_ = 0.21, 95% CI [− 0.67, 1.10]). Table 1 presents the means and 95% CIs of all groups and comparisons with the composite, objective and subjective scores separately.

**Table 1:** The mean composite, objective and subjective SWASH Scores with 95% CIs broken down by the type of the second screen and the length of the delay

Group	Measure		
	Composite	Objective	Subjective
Offline	3.44 [2.73, 4.16]	3.92 [3.17, 4.67]	2.96 [2.19, 3.73]
Online	3.13 [2.54, 3.71]	3.53 [2.89, 4.17]	2.72 [2.13, 3.32]
Difference	0.31 [− 0.59, 1.22]	0.39 [− 0.58, 1.36]	0.24 [− 0.72, 1.19]
Short delay	3.32 [2.72, 3.91]	3.89 [3.26, 4.52]	2.74 [2.14, 3.35]
Long delay	3.10 [2.42, 3.78]	3.28 [2.54, 4.02]	2.93 [2.19, 3.67]
Difference	0.21 [− 0.67, 1.10]	0.61 [− 0.34, 1.57]	− 0.19 [− 1.12, 0.75]

Values within the squared brackets represent the 95% confidence intervals. Data presented in this table are based solely on the second screen

### Validity

The correlation between the objective and subjective subscales of the SWASH was strong for the offline screen (*r* = .78, 95% CI [0.56, 0.89]) as well as for the online screen (*r* = .79, 95% CI [0.65, 0.88]) indicating appropriate validity in this respect. The difference between the offline and online screen in terms of the strength of the correlation between the objective and subjective scales was close to zero (*r* = − .02, 95% CI [− .25, 0.17]).

### Test–retest reliability (non-preregistered)

Correlation between the first and the second screen scores was strong for the subjective subscale, but only moderate for the objective subscale irrespective of the type of the screen. For the composite scores, the correlation was strong for the online and offline group as well indicating a good enough test–retest reliability of the SWASH. Interestingly, the test–retest reliability of the online group was possibly higher than that of the offline group, although, only to a small extent (see Table 2 for *r*s and their 95% CIs). The correlation between the first and second screen scores was strong in the short delay group for the subscales as well as for the composite scores. However, the correlation was only moderate in the long delay group implying that the test–retest reliability of the SWASH is influenced by the length of the delay between the screens from a weak to a moderate extent. Table 2 presents the exact correlation values and their 95% CIs separately for the experimental groups.

**Table 2:** Test–retest reliability of SWASH Scores broken down by type of screen and length of delay

Group	Measure		
	Composite	Objective	Subjective
Offline	0.62 [0.31, 0.81]	0.43 [0.05, 0.70]	0.69 [0.42, 0.85]
Online	0.74 [0.57, 0.85]	0.59 [0.35, 0.75]	0.77 [0.61, 0.87]
Difference	− 0.12 [− .45, 0.14]	− 0.16 [− 0.57, 0.20]	− 0.07 [− 0.37, 0.15]
Short delay	0.79 [0.65, 0.88]	0.65 [0.44, 0.79]	0.81 [0.68, 0.89]
Long delay	0.55 [0.20, 0.78]	0.37 [− 0.03, 0.67]	0.56 [0.21, 0.78]
Difference	0.24 [− 0.02, 0.61]	0.28 [− 0.08, 0.70]	0.25 [− 0.01, 0.61]

The correlation values are all Pearson’s *r*s and the 95% CIs are reported within the squared brackets

### Depth of hypnosis

#### Difference between the groups

**Fig. 2:**
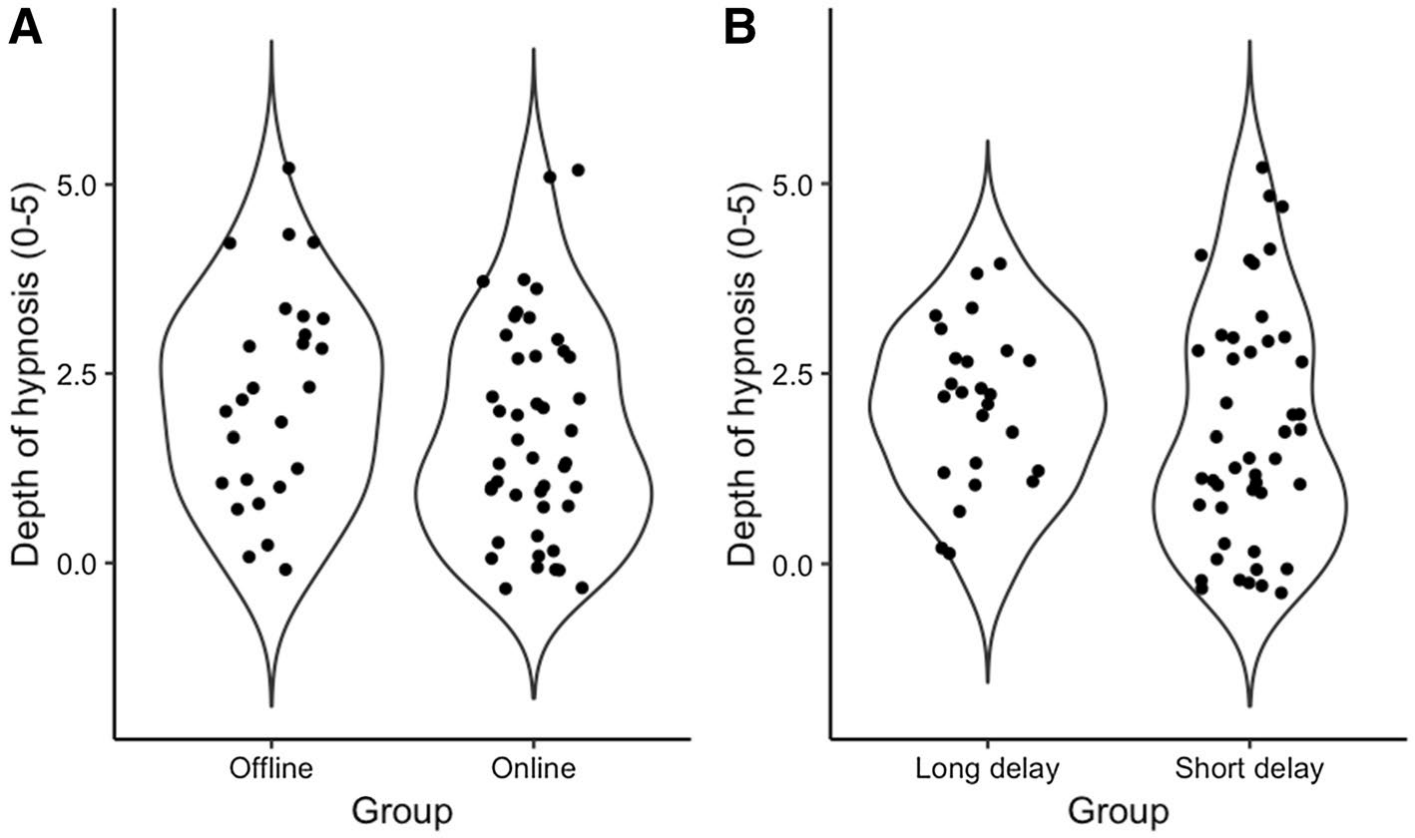
Violin plots representing the distribution of depth of hypnosis scores separately for the offline and online screens (**a**), and for the short and long delay groups (**b**)

The participants reported somewhat higher depth of hypnosis scores in the offline (*M* = 2.15, 95% CI [1.61, 2.70]) than in the online (*M* = 1.73, 95% CI [1.31, 2.16]) group. Nonetheless, the difference between the groups is not substantial and the maximum plausible value of this difference is 1.10 (*M* = 0.42, 95% CI [-0.26, 1.10]). The mean of the depth of hypnosis scores in the short delay (*M* = 1.80, 95% CI [1.35–2.26]) compared to the long delay group (*M* = 2.04, 95% CI [1.59, 2.49]) differed only to a small extent (*M* = − 0.24, 95% CI [− 0.87, 0.40]). Figure 2 portrays the distribution of the depth of hypnosis scores broken down by the type of the second screen (panel A) and the length of the delay between the first and second screen (panel B). The depth of hypnosis scores are similarly distributed in the offline and online groups.

#### Correlation between SWASH and depth of hypnosis scores (non-preregistered)

The correlation between the SWASH and depth of hypnosis scores was strong for all but one measure in the online and for all in the offline screen group (all *r* > .54). The strength of the correlation is unlikely to be larger than 0.21 in the offline group than in the online group rendering the difference between the two groups minimal. There was strong correlation between the depth of hypnosis scores and all measures in the short delay group (all *r* > .70), and the correlations were moderate to strong in the long delay group (all *r* > .31). The difference between the two groups for the strength of the correlations was weak to moderate, and it was the highest for the objective scores. Table 3 shows all of the correlation values and their 95% CIs separately for the experimental groups and for all of the measures.

**Table 3:** Correlation between SWASH and depth of hypnosis scores broken down by the type of screen and the length of delay

Group	Measure		
	Composite	Objective	Subjective
Offline	0.76 [0.53, 0.89]	0.66 [0.37, 0.84]	0.77 [0.54, 0.89]
Online	0.70 [0.52, 0.83]	0.54 [0.29, 0.72]	0.81 [0.67, 0.89]
Difference	0.06 [− 0.21, 0.28]	0.12 [− 0.22, 0.43]	− 0.04 [− 0.28, 0.15]
Short delay	0.79 [0.65, 0.88]	0.70 [0.51, 0.82]	0.83 [0.71, 0.90]
Long delay	0.55 [0.20, 0.78]	0.31 [− 0.10, 0.63]	0.70 [0.41, 0.86]
Difference	0.24 [− 0.03, 0.60]	0.38 [0.02, 0.81]	0.13 [− 0.07, 0.42]

The correlation values are all Pearson’s *r*s and the 95% CIs are reported within the squared brackets

## Discussion

The purpose of the present study was to explore whether online hypnosis screening is feasible as the adaptation of this method could ease the recruitment-related costs of hypnosis research. To this aim, we estimated the extent to which offline and online hypnosis screening scores, measured by the SWASH, are comparable. The results revealed that the difference between offline and online groups was small to negligible in all aspects and, importantly, applying online rather than offline screening is unlikely to reduce the composite screening score by more than 1.22 and the objective score by more than 1.36 out of ten. To put these effect sizes in perspective, for instance, a recent meta-analysis of four studies investigating the influence of standard induction procedures on suggestibility found that, on average, people score 1.46 higher (out of ten) on scales assessing objective responses to suggestions if they had received a priori induction compared to no induction (Martin & Dienes, 2019). Moreover, the average SWASH score in the online group was comparable to the result of an earlier screen conducted in group sessions at the same university (Lush et al., 2018). Finally, it is not only the average scores in the online group that can be deemed acceptable, the distribution of SWASH scores were also akin in the offline and online groups even at the positive end of the scale. This implies that some people can successfully respond to many suggestions when they undertake an online screening (see Fig. 1). None of this was obvious before the data were collected.

The correlation between objective and subjective scores was strong for both of the offline and online groups; crucially, the correlation in the online group can only be as small as 0.65. This indicates that the validity of the SWASH remained acceptable even with online data collection. Moreover, the strength of the correlation between the subjective and objective components of the SWASH found by Lush et al. (2018) was 0.70, which is consistent with our results. The strength of the correlation between SWASH scores of the first and second screens was medium in the offline and strong in the online group. The lower bound of the 95% CI in the online group was 0.57 implying that the test–retest reliability of the online measurement is adequate. These values are also appropriate in relative terms. For instance, Fassler et al. (2008) employed the CURSS which has an objective and a subjective subscale such as the SWASH, in two occasions and the test–retest correlations were 0.59 and 0.77 for the objective and subjective components, respectively. These results are in line with the correlations found by us in the online group. Overall, the psychometric properties of online screening were excellent; the quality of data collected online has shown to be consistent with the quality of offline data gathered within this study and as part of earlier studies with the SWASH and other hypnosis screening tools.

Modern theories of hypnosis advocate the notion that all hypnosis is self-hypnosis, since the hypnotic subject is the one who actively responds to the suggestions and creates the requested experience (Kihlstrom, 2008; Raz, 2011). This does not mean, however, that the experimenter has no influence on the responsiveness of the subject. For instance, the presence of an experimenter can be helpful in building up a rapport and facilitating responsiveness of the participants (e.g. Gfeller, Lynn, & Pribble, 1987). Nonetheless, the experimenter can also bias the responses of the subjects (e.g. Barber & Calverley, 1966; Troffer & Tart, 1964), and importantly, this level of bias can strongly vary across participants as it is almost impossible to deliver the induction and suggestions in an identical way multiple times. Therefore, the application of fully automatised screenings, such as the online version, can subserve the standardisation of the assessment of hypnotic suggestibility.

Introducing online hypnosis screening would markedly decrease the amount of time experimenters need to invest to find participants for their studies. However, to complete a screening procedure, the participants still need to spend 45–60 min without taking a break; otherwise, the data would be not usable for recruitment purposes. A substantial part of the screening is assigned to the standard hypnotic induction, which consists of various suggestions mostly to relax; however, the responses to these suggestions are not assessed directly during the screening (e.g. Shor & Orne, 1963; Weitzenhoffer & Hilgard, 1962). Would it be feasible to exclude the standard induction from the screening procedure to save time for the participants? Cognitive theories of hypnosis, such as the cold control theory (Barnier, Dienes, & Mitchell, 2008; Dienes & Perner, 2007), emphasise the role of the feeling of involuntariness in differentiating hypnotic from non-hypnotic responses. This feeling is also known as the “classical suggestion effect” (Weitzenhoffer, 1974, 1980). Therefore, according to cold control theory, not the practice of induction, but the feeling of involuntariness is the demarcation criterion, and it is important to ensure with self-report measures that the participants experienced a reduction in the level of control over their own behaviour (e.g. Palfi, Parris, McLatchie, Kekecs, & Dienes, 2018)^1^. From a practical perspective, it is important to bear in mind that the presence of a standard induction can increase responsiveness to the suggestions in the screening, on average, by 1.46 (Martin & Dienes, 2019) compared to the absence of the induction; and that the strength of the effect of an induction fluctuates across suggestions (Terhune & Cardeña, 2016). Nonetheless, as argued earlier in this paper, a general reduction of responsiveness does not qualify as decisive argument for retaining the induction procedure. As long as the absence of the induction does not produce a floor-effect or alters markedly the ranking of the suggestibility scores, the screening can be perfectly adequate for screening people for individual differences in response. Indeed, there are existing attempts to assess responsiveness to suggestions without exposing the participants to an induction, such as the Barber Suggestibility Scale (Barber & Glass, 1962) and the CIS (Wilson & Barber, 1978). These scales can be easily administered in a context presented as a test of imagination while applying motivational instructions to replace the induction or simply leaving out the induction. The existing evidence suggests that employing motivational instructions creates similar level of responsiveness as the application of the induction; however, the absence of the induction significantly dwindles the level of responsiveness to suggestions (Barber & Wilson, 1978). Future research could explore the extent to which the exclusion or replacement of the induction from the SWASH would be feasible and assess whether it would be beneficial.

A secondary interest of the current study was to assess the extent to which the length of the delay between the first and second screening affects the outcome of the screen and the psychometric properties of the measurement tool. Repeated assessment of suggestibility can negatively affect the suggestibility scores, for instance, if the delay amid the two occasions takes only a few days or weeks (Barber & Calverley, 1966; Fassler et al., 2008; Lynn et al., 1988). This reduction in suggestibility may be caused by boredom; the participants can become disengaged with the procedure by virtue of finding it repetitive (Barber & Calverley, 1966; Fassler et al., 2008). In our case, the short delay was a minimum of 5 months and we found no indication of substantial differences between the short and long delay groups among the SWASH subscales. For instance, Fassler et al. (2008) found a difference of 0.77 on the objective scores between the first and second session^2^, but according to our data, the largest plausible difference is only 0.34. Nonetheless, the effect of boredom on the subjective scores observed by Fassler et al. (2008) was 1.05,^3^ which is compatible with our results as the lower bound of the difference in that aspect was 1.12. Taken together, our data imply that the negative effect of boredom might wear off or becomes negligible after 5 months; however, more research is needed to settle this matter and identify the ideal amount of delay that can prevent boredom effects in repeated designs.

We note that our sample was restricted to university students, which might preclude the generalisation of our findings, crucially, the applicability of online hypnosis screening, to a wider population. Nonetheless, the problem of generalisability represents a universal issue in experimental hypnosis research. For instance, a meta-analysis on 27 studies investigating hypnotically induced analgesia found that from the studies with non-clinical samples (*N* = 19), only one was run with people recruited from the local community whereas all the other studies were run with students (Montgomery, Duhamel, & Redd, 2000). Recruiting from a wider population would not only increase generalisability of the findings, but it would further facilitate researchers to run large-scale hypnosis studies strengthening the replicability of the findings. Future research is needed to explore the extent to which online hypnosis research can be applied to screen and recruit people from local communities.

Finally, the vast majority of our participants were females; hence, the gender imbalance in our sample might be another factor hindering the generalisability of our findings. Research on the link between gender and hypnotic suggestibility has provided ambiguous results with some studies finding virtually no effect (Cooper & London, 1966; Dienes, Brown, Hutton, Kirsch, Mazzoni, & Wright, 2009; McConkey, Barnier, Maccallum, & Bishop, 1996) and some studies demonstrating a small effect size (Green, 2004; Green & Lynn, 2010; Morgan & Hilgard, 1973; Page & Green, 2007; Rudski, Marra, & Graham, 2004). Studies showing a small effect size of gender consistently found that women score higher than men, which might be caused by a divergence in a personality trait that partly underlies suggestibility or difference between women and men in how they assess the difficulty of the suggestions (Rudski, Marra, & Graham, 2004). Nonetheless, these explanations are conjectures that have yet to be tested. With only seven men in the current data set, we can only speculate how much gender might moderate the difference the online compared to the offline measurement of hypnotic suggestibility.

## Conclusion

Altogether, the online assessment of hypnotic suggestibility appears to be feasible and the benefits far outweigh the downsides involved with its application. Although, online screening might be less engaging than the traditional, offline measurement of suggestibility and so it can result in slightly lower suggestibility scores, our study suggests that the effect size of this negative impact lies within acceptable boundaries. Crucially, the application of online hypnosis screening can subserve the execution of large-scale data collection with heterogeneous samples consisting of student and non-student participants as well. Furthering our knowledge based on small sample studies comes with many risks (e.g. Loken & Gelman, 2017), but the relative high cost of hypnosis screening procedures hinders the researchers of the field from running well-powered studies. Therefore, we argue that the adaptation of online hypnosis screening is salutary and it helps experimental hypnosis research to realise its full potentials.

## Footnotes

1 An operational definition of hypnosis necessitates the usage of induction to render suggestions hypnotic, and labels all suggestions without a priori induction imaginative suggestion (Braffman & Kirsch, 1999; Kirsch, 1997; Kirsch & Braffman, 2001). This line of thinking would preclude us from omitting the induction in case we want to measure hypnotic suggestibility.

2 The reported raw difference was 0.54; however, we adjusted this value from a scale of 0–7 to the scale of the SWASH, which is 0–10.

3 The reported raw difference was 2.2; we adjusted this value as well from a scale of 0–21 to the scale of the SWASH in which values can vary between 0 and 10.
